# Partial cone conformer of 25,27-bis­[(methoxy­carbonyl)­meth­oxy]-26,28-dipropoxycalix[4]arene

**DOI:** 10.1107/S1600536808006843

**Published:** 2008-03-14

**Authors:** Guo-Zhi Zhang, Mei Zhao, Xiao-Ling Zhang, Jian-Ping Ma, Dian-Shun Guo

**Affiliations:** a Department of Chemistry, Shandong Normal University, Jinan 250014, People’s Republic of China

## Abstract

Mol­ecules of the title compound, C_40_H_44_O_8_, adopt a partial cone conformation. The dihedral angles between the planes of the aromatic rings and the mean plane through the methyl­ene C atoms bridging the aromatic rings are 35.74 (7), 85.86 (5), 87.77 (4) and 89.95 (5)°. Two opposite aryl rings are approximately parallel to each other; the others are at an inter­planar angle of 52.41 (6)°. Intra- and inter­molecular C—H⋯O hydrogen bonds stabilize the mol­ecular conformation and the crystal packing. Two C atoms of one propoxy chain are disordered over two positions; the site occupancy factors are *ca* 0.66 and 0.34.

## Related literature

For related literature, see: Arena *et al.* (1997 ▶); Ferguson *et al.* (1993 ▶); Gutsche (1998 ▶); Iwamoto & Shinkai (1992 ▶); Pappalardo *et al.* (1992 ▶); Yamato *et al.* (1998 ▶).
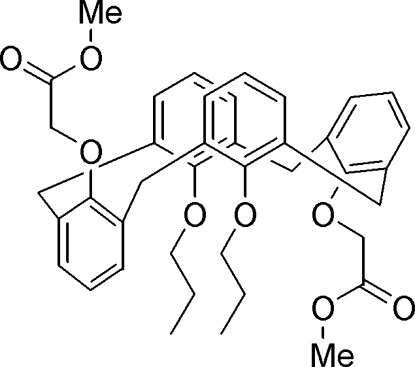


## Experimental

### 

#### Crystal data

C_40_H_44_O_8_
*M* *_r_* = 652.75
Monoclinic, 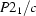

*a* = 9.3007 (18) Å
*b* = 18.114 (4) Å
*c* = 20.768 (4) Å
β = 101.334 (3)°
*V* = 3430.6 (11) Å^3^
*Z* = 4
Mo *K*α radiation
μ = 0.09 mm^−1^
*T* = 298 (2) K
0.37 × 0.18 × 0.08 mm

#### Data collection

Bruker SMART CCD area-detector diffractometer
Absorption correction: none
17798 measured reflections
6362 independent reflections
4677 reflections with *I* > 2σ(*I*)
*R*_int_ = 0.038

#### Refinement

*R*[*F* ^2^ > 2σ(*F* ^2^)] = 0.052
*wR*(*F* ^2^) = 0.113
*S* = 1.04
6362 reflections
457 parameters
1 restraint
H-atom parameters constrained
Δρ_max_ = 0.23 e Å^−3^
Δρ_min_ = −0.21 e Å^−3^

### 

Data collection: *SMART* (Bruker, 1999 ▶); cell refinement: *SAINT* (Bruker, 1999 ▶); data reduction: *SAINT*; program(s) used to solve structure: *SHELXS97* (Sheldrick, 2008 ▶); program(s) used to refine structure: *SHELXL97* (Sheldrick, 2008 ▶); molecular graphics: *SHELXTL* (Sheldrick, 2008 ▶); software used to prepare material for publication: *SHELXTL*.

## Supplementary Material

Crystal structure: contains datablocks I, global. DOI: 10.1107/S1600536808006843/bt2681sup1.cif

Structure factors: contains datablocks I. DOI: 10.1107/S1600536808006843/bt2681Isup2.hkl

Additional supplementary materials:  crystallographic information; 3D view; checkCIF report

## Figures and Tables

**Table 1:** Hydrogen-bond geometry (Å, °)

*D*—H⋯*A*	*D*—H	H⋯*A*	*D*⋯*A*	*D*—H⋯*A*
C30—H30*B*⋯O8^i^	0.96	2.58	3.406 (3)	144
C31—H31*B*⋯O5	0.97	2.40	2.859 (2)	109
C24—H24⋯O3^ii^	0.93	2.58	3.309 (3)	135
C21—H21*A*⋯O4	0.97	2.45	2.899 (2)	108
C21—H21*A*⋯O2	0.97	2.56	3.255 (3)	129
C10—H10*C*⋯O8^iii^	0.96	2.59	3.510 (3)	161

Symmetry codes: (i) 
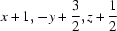
; (ii) 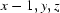; (iii) 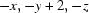.

**Fig. 1.**
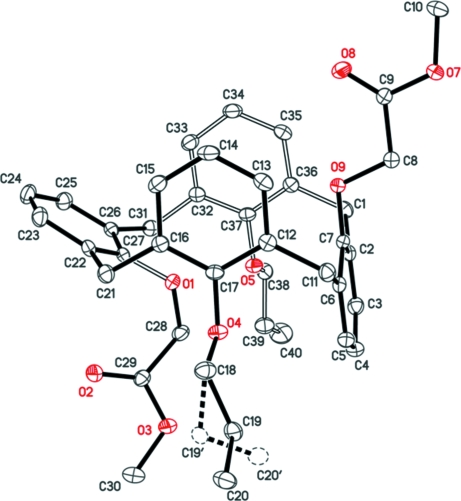
The molecular structure of the title compound, showing the atomic numbering scheme and displacement ellipsoids drawn at the 30% probability level for non-H atoms. The major occupied site of the disordered side chain is drawn with dashed bonds.

## supplementary crystallographic information

### Comment

Substituted calix[4]arenes, as the most fascinating macrocyclic receptors in supramolecular chemistry, have attracted much interest in recent years due to the high affinity and ion selectivity (Gutsche, 1998). In particular, much attention was paid to the ethyl ester derivatives of the calix[4]arene (Iwamoto & Shinkai, 1992; Arena *et al.*, 1997) since they are one of the most versatile intermediates for building highly preorganized receptors. It has been shown that the conformational distribution in the exhaustive *O*-alkylation of the calix[4]arene depends upon the reaction conditions, the *para* substituent of the calix[4]arene, and the steric requirement of the agent (Pappalardo *et al.*, 1992; Ferguson *et al.*, 1993). In the *O*-alkylation, the cone and 1,3-alternate conformers are often obtained *via* the metal template effect using potassium and caesium ions, respectively (Yamato *et al.*, 1998). However, both partial cone and cone conformers were isolated instead of the 1,3-alternate conformer when treatment 25,27-dihydroxy-26,28-dipropoxycalix[4]arene with methyl bromoacetate using caesium carbonate as a base in acetone.

The title calix[4]arene compound adopts a partial cone conformation (Ferguson *et al.*, 1993) (Fig. 1). The dihedral angles between the planes of the aromatic rings and the mean plane through the methylene C atoms bridging the aromatic rings are 35.74 (7), 85.86 (5), 87.77 (4) and 89.95 (5)°. The C2–C7 and C22–C27 rings make an interplanar angle of 52.41 (6)°. However the C12–C17 and C32–C37 rings are almost parallel to each other, with a dihedral angle of 4.17 (11)°. The O···O separations of ethereal O atoms are O1···O4 3.017 (2), O1···O5 3.365 (2), O4···O9 4.693 (2), and O5···O9 4.588 (2) Å. This conformation precludes any solvent molecule being enclathrated within the small molecular cavity.

In the crystal structure there are intra- and intermolecular C—H···O hydrogen bonds (Table 1), which stabilize the partial cone conformation and the crystal packing.

### Experimental

A mixture of 25,27-dihydroxy-26,28-dipropoxycalix[4]arene (0.200 g, 0.39 mmol), anhydrous caesium carbonate (0.190 g, 0.59 mmol) and methyl bromoacetate (0.11 ml, 1.17 mmol) in dry acetone (10 ml) was refluxed under nitrogen for 8 h and cooled to room temperature. After removal of the solvent under reduced pressure, the residue was treated with 5% aqueous hydrochloric acid and extracted with dichloromethane. The organic layer was washed with saturated sodium hydrogen carbonate and brine, dried over anhydrous magnesium sulfate. The solvent was removed under reduced pressure and the residue was purified by flash column (silica gel, EtOAc/petroleum ether = 1:5) to give conformer (I) (*R*_f _= 0.7) and comformer (II) (*R*_f _= 0.4) in 48% and 39% yields, respectively. Single crystals suitable for X-ray diffraction analysis were obtained by slow diffusion of methanol into a dichloromethane solution at 273 K.

### Refinement

Hydrogen atoms were placed in geometrically idealized positions and refined using a riding model with C_aromatic_—H = 0.93Å [*U*_iso_(H) = 1.2*U*_eq_(C)], C_methylene_—H = 0.96Å [*U*_iso_(H) = 1.2*U*_eq_(C)] or C_methyl_—H = 0.97Å [*U*_iso_(H) = 1.5*U*_eq_(C)]. Atoms C19 and C20 are disordered over two positions (C19/C19' and C20/C20'), with refined occupancies of 0.337 (5) and 0.663 (5). One of the two CH_2_—CH_3_ distances of the disordered C-atoms (C18—C19) was restrained to 1.54 (1) Å.

### Crystal data

**Table:**

C_40_H_44_O_8_	*F*_000_ = 1392
*M**_r_* = 652.75	*D*_x_ = 1.264 Mg m^−^^3^
Monoclinic, *P*2_1_/*c*	Mo *K*α radiation λ = 0.71073 Å
*a* = 9.3007 (18) Å	Cell parameters from 4185 reflections
*b* = 18.114 (4) Å	θ = 2.3–28.2º
*c* = 20.768 (4) Å	µ = 0.09 mm^−^^1^
β = 101.334 (3)º	*T* = 298 (2) K
*V* = 3430.6 (11) Å^3^	Block, colourless
*Z* = 4	0.37 × 0.18 × 0.08 mm

### Data collection

**Table:**

Bruker SMART CCD area-detector diffractometer	4677 reflections with *I* > 2σ(*I*)
Radiation source: fine-focus sealed tube	*R*_int_ = 0.038
Monochromator: graphite	θ_max_ = 25.5º
*T* = 298(2) K	θ_min_ = 2.0º
φ and ω scans	*h* = −11→10
Absorption correction: none	*k* = −21→21
17798 measured reflections	*l* = −25→21
6362 independent reflections	

### Refinement

**Table:**

Refinement on *F*^2^	Secondary atom site location: difference Fourier map
Least-squares matrix: full	Hydrogen site location: inferred from neighbouring sites
*R*[*F*^2^ > 2σ(*F*^2^)] = 0.052	H-atom parameters constrained
*wR*(*F*^2^) = 0.113	*w* = 1/[σ^2^(*F*_o_^2^) + (0.0427*P*)^2^ + 0.8294*P*] where *P* = (*F*_o_^2^ + 2*F*_c_^2^)/3
*S* = 1.04	(Δ/σ)_max_ = 0.001
6362 reflections	Δρ_max_ = 0.23 e Å^−^^3^
457 parameters	Δρ_min_ = −0.21 e Å^−^^3^
1 restraint	Extinction correction: none
Primary atom site location: structure-invariant direct methods	

### Special details

**Table:**

Geometry. All e.s.d.'s (except the e.s.d. in the dihedral angle between two l.s. planes) are estimated using the full covariance matrix. The cell e.s.d.'s are taken into account individually in the estimation of e.s.d.'s in distances, angles and torsion angles; correlations between e.s.d.'s in cell parameters are only used when they are defined by crystal symmetry. An approximate (isotropic) treatment of cell e.s.d.'s is used for estimating e.s.d.'s involving l.s. planes.
Refinement. Refinement of *F*^2^ against ALL reflections. The weighted *R*-factor *wR* and goodness of fit *S* are based on *F*^2^, conventional *R*-factors *R* are based on *F*, with *F* set to zero for negative *F*^2^. The threshold expression of *F*^2^ > σ(*F*^2^) is used only for calculating *R*-factors(gt) *etc*. and is not relevant to the choice of reflections for refinement. *R*-factors based on *F*^2^ are statistically about twice as large as those based on *F*, and *R*- factors based on ALL data will be even larger.

### Fractional atomic coordinates and isotropic or equivalent isotropic displacement parameters (Å^2^)

**Table:**

	*x*	*y*	*z*	*U*_iso_*/*U*_eq_	Occ. (<1)
C1	0.4972 (2)	0.73848 (11)	0.02361 (9)	0.0283 (5)	
H1A	0.4664	0.7738	−0.0115	0.034*	
H1B	0.5528	0.7000	0.0071	0.034*	
C2	0.5945 (2)	0.77685 (10)	0.08095 (9)	0.0246 (4)	
C3	0.7334 (2)	0.75018 (11)	0.10761 (10)	0.0301 (5)	
H3	0.7710	0.7106	0.0876	0.036*	
C4	0.8167 (2)	0.78136 (11)	0.16329 (10)	0.0326 (5)	
H4	0.9105	0.7637	0.1801	0.039*	
C5	0.7595 (2)	0.83898 (11)	0.19370 (9)	0.0288 (5)	
H5	0.8147	0.8588	0.2320	0.035*	
C6	0.6214 (2)	0.86838 (10)	0.16860 (9)	0.0237 (4)	
C7	0.5431 (2)	0.83749 (10)	0.11086 (9)	0.0230 (4)	
C8	0.4144 (2)	0.92188 (11)	0.03600 (10)	0.0297 (5)	
H8A	0.4717	0.9041	0.0049	0.036*	
H8B	0.4629	0.9651	0.0578	0.036*	
C9	0.2634 (2)	0.94198 (11)	0.00053 (9)	0.0268 (5)	
C10	0.1375 (2)	1.02558 (13)	−0.07828 (11)	0.0430 (6)	
H10A	0.1019	0.9897	−0.1116	0.065*	
H10B	0.1550	1.0714	−0.0986	0.065*	
H10C	0.0659	1.0329	−0.0513	0.065*	
C11	0.5597 (2)	0.93063 (10)	0.20352 (10)	0.0277 (5)	
H11A	0.6329	0.9458	0.2411	0.033*	
H11B	0.5394	0.9725	0.1740	0.033*	
C12	0.4208 (2)	0.90960 (10)	0.22657 (9)	0.0244 (4)	
C13	0.2855 (2)	0.93849 (11)	0.19661 (9)	0.0292 (5)	
H13	0.2814	0.9736	0.1637	0.035*	
C14	0.1574 (2)	0.91623 (11)	0.21461 (10)	0.0328 (5)	
H14	0.0685	0.9375	0.1951	0.039*	
C15	0.1615 (2)	0.86226 (11)	0.26167 (9)	0.0294 (5)	
H15	0.0743	0.8459	0.2724	0.035*	
C16	0.2935 (2)	0.83180 (10)	0.29339 (9)	0.0239 (4)	
C17	0.4221 (2)	0.85832 (10)	0.27674 (9)	0.0232 (4)	
C18	0.6063 (2)	0.87236 (11)	0.36982 (10)	0.0322 (5)	
H18A	0.5593	0.8549	0.4047	0.039*	0.337 (5)
H18B	0.5881	0.9248	0.3634	0.039*	0.337 (5)
C19	0.7747 (8)	0.8551 (5)	0.3847 (4)	0.0331 (19)	0.337 (5)
H19A	0.7915	0.8026	0.3919	0.040*	0.337 (5)
H19B	0.8190	0.8704	0.3483	0.040*	0.337 (5)
C20	0.8404 (8)	0.8985 (4)	0.4468 (3)	0.044 (2)	0.337 (5)
H20A	0.8208	0.9502	0.4393	0.067*	0.337 (5)
H20B	0.9444	0.8906	0.4574	0.067*	0.337 (5)
H20C	0.7970	0.8820	0.4826	0.067*	0.337 (5)
C21	0.2928 (2)	0.76960 (11)	0.34205 (9)	0.0287 (5)	
H21A	0.3929	0.7582	0.3632	0.034*	
H21B	0.2407	0.7855	0.3758	0.034*	
C22	0.2207 (2)	0.70070 (10)	0.30918 (9)	0.0256 (5)	
C23	0.0867 (2)	0.67571 (11)	0.32014 (10)	0.0327 (5)	
H23	0.0414	0.7006	0.3499	0.039*	
C24	0.0191 (2)	0.61448 (12)	0.28763 (11)	0.0359 (5)	
H24	−0.0697	0.5977	0.2963	0.043*	
C25	0.0841 (2)	0.57875 (11)	0.24254 (10)	0.0318 (5)	
H25	0.0380	0.5378	0.2206	0.038*	
C26	0.2170 (2)	0.60219 (10)	0.22883 (9)	0.0250 (4)	
C27	0.2867 (2)	0.66187 (10)	0.26456 (9)	0.0246 (4)	
C28	0.5503 (2)	0.65411 (12)	0.27113 (9)	0.0310 (5)	
H28A	0.6263	0.6816	0.2555	0.037*	
H28B	0.5433	0.6060	0.2503	0.037*	
C29	0.5965 (2)	0.64378 (11)	0.34452 (10)	0.0282 (5)	
C30	0.8024 (3)	0.63060 (15)	0.42966 (11)	0.0500 (7)	
H30A	0.7630	0.6642	0.4575	0.075*	
H30B	0.9071	0.6356	0.4374	0.075*	
H30C	0.7774	0.5809	0.4392	0.075*	
C31	0.2738 (2)	0.56959 (10)	0.17147 (9)	0.0283 (5)	
H31A	0.2203	0.5247	0.1570	0.034*	
H31B	0.3766	0.5569	0.1854	0.034*	
C32	0.2563 (2)	0.62392 (10)	0.11495 (9)	0.0258 (5)	
C33	0.1197 (2)	0.65398 (12)	0.08963 (10)	0.0337 (5)	
H33	0.0381	0.6377	0.1052	0.040*	
C34	0.1031 (2)	0.70765 (12)	0.04169 (10)	0.0352 (5)	
H34	0.0106	0.7267	0.0245	0.042*	
C35	0.2239 (2)	0.73306 (11)	0.01923 (9)	0.0305 (5)	
H35	0.2121	0.7699	−0.0125	0.037*	
C36	0.3630 (2)	0.70474 (10)	0.04306 (9)	0.0250 (5)	
C37	0.3764 (2)	0.64870 (10)	0.09015 (9)	0.0247 (4)	
C38	0.5595 (2)	0.56049 (11)	0.08040 (10)	0.0312 (5)	
H38A	0.5730	0.5776	0.0377	0.037*	
H38B	0.4847	0.5224	0.0737	0.037*	
C39	0.7012 (2)	0.52970 (12)	0.11846 (11)	0.0407 (6)	
H39A	0.6836	0.5077	0.1588	0.049*	
H39B	0.7707	0.5697	0.1303	0.049*	
C40	0.7671 (3)	0.47225 (15)	0.07997 (13)	0.0624 (8)	
H40A	0.6979	0.4330	0.0673	0.094*	
H40B	0.8548	0.4527	0.1068	0.094*	
H40C	0.7905	0.4946	0.0414	0.094*	
H18C	0.5279	0.8738	0.3944	0.075*	0.663 (5)
H18D	0.6276	0.9227	0.3589	0.075*	0.663 (5)
C19'	0.7406 (4)	0.8397 (2)	0.41222 (19)	0.0274 (9)	0.663 (5)
H19C	0.7202	0.7895	0.4240	0.033*	0.663 (5)
H19D	0.7671	0.8681	0.4524	0.033*	0.663 (5)
C20'	0.8681 (4)	0.8398 (2)	0.37574 (16)	0.0386 (10)	0.663 (5)
H20D	0.8457	0.8075	0.3384	0.058*	0.663 (5)
H20E	0.9554	0.8229	0.4046	0.058*	0.663 (5)
H20F	0.8832	0.8889	0.3612	0.058*	0.663 (5)
O1	0.41503 (14)	0.69143 (7)	0.25091 (6)	0.0268 (3)	
O2	0.51744 (17)	0.63185 (10)	0.38196 (7)	0.0542 (5)	
O3	0.74159 (15)	0.64726 (9)	0.36180 (7)	0.0397 (4)	
O4	0.55748 (14)	0.83184 (7)	0.31032 (6)	0.0275 (3)	
O5	0.51464 (14)	0.62042 (7)	0.11647 (6)	0.0281 (3)	
O7	0.27257 (15)	0.99957 (8)	−0.03810 (7)	0.0366 (4)	
O8	0.15084 (17)	0.91286 (9)	0.00548 (8)	0.0484 (4)	
O9	0.40569 (14)	0.86619 (7)	0.08311 (6)	0.0251 (3)	

### Atomic displacement parameters (Å^2^)

**Table:**

	*U*^11^	*U*^22^	*U*^33^	*U*^12^	*U*^13^	*U*^23^
C1	0.0350 (13)	0.0289 (11)	0.0214 (11)	0.0030 (9)	0.0069 (9)	−0.0009 (8)
C2	0.0250 (12)	0.0298 (11)	0.0205 (10)	−0.0020 (9)	0.0079 (8)	0.0026 (8)
C3	0.0294 (13)	0.0302 (12)	0.0325 (12)	0.0026 (9)	0.0106 (10)	0.0014 (9)
C4	0.0236 (12)	0.0392 (13)	0.0338 (12)	0.0045 (9)	0.0029 (9)	0.0064 (10)
C5	0.0255 (12)	0.0356 (12)	0.0237 (11)	−0.0040 (9)	0.0009 (9)	0.0018 (9)
C6	0.0229 (11)	0.0256 (11)	0.0233 (11)	−0.0044 (8)	0.0061 (8)	0.0038 (8)
C7	0.0181 (11)	0.0276 (11)	0.0236 (11)	−0.0006 (8)	0.0051 (8)	0.0062 (8)
C8	0.0246 (12)	0.0328 (12)	0.0315 (12)	−0.0004 (9)	0.0050 (9)	0.0081 (9)
C9	0.0239 (12)	0.0282 (11)	0.0289 (12)	0.0000 (9)	0.0065 (9)	0.0028 (9)
C10	0.0336 (14)	0.0488 (14)	0.0409 (14)	0.0029 (11)	−0.0070 (11)	0.0170 (11)
C11	0.0312 (13)	0.0252 (11)	0.0265 (11)	−0.0046 (9)	0.0051 (9)	0.0004 (8)
C12	0.0280 (12)	0.0218 (10)	0.0232 (11)	0.0009 (8)	0.0046 (9)	−0.0039 (8)
C13	0.0345 (14)	0.0264 (11)	0.0254 (11)	0.0058 (9)	0.0032 (9)	0.0028 (8)
C14	0.0238 (12)	0.0398 (13)	0.0318 (12)	0.0089 (9)	−0.0022 (9)	0.0002 (10)
C15	0.0214 (12)	0.0364 (12)	0.0306 (12)	−0.0014 (9)	0.0058 (9)	−0.0045 (9)
C16	0.0247 (12)	0.0256 (10)	0.0216 (10)	0.0004 (8)	0.0046 (8)	−0.0044 (8)
C17	0.0219 (11)	0.0229 (10)	0.0237 (11)	0.0050 (8)	0.0020 (8)	−0.0025 (8)
C18	0.0298 (13)	0.0291 (11)	0.0342 (12)	−0.0016 (9)	−0.0023 (9)	−0.0022 (9)
C19	0.034 (6)	0.047 (5)	0.019 (5)	0.002 (4)	0.009 (4)	0.000 (3)
C20	0.041 (5)	0.051 (5)	0.034 (4)	−0.007 (3)	−0.009 (3)	0.011 (3)
C21	0.0304 (13)	0.0338 (12)	0.0234 (11)	−0.0001 (9)	0.0084 (9)	−0.0020 (9)
C22	0.0293 (12)	0.0281 (11)	0.0200 (10)	0.0001 (9)	0.0064 (9)	0.0051 (8)
C23	0.0343 (14)	0.0351 (12)	0.0320 (12)	0.0018 (10)	0.0149 (10)	0.0020 (9)
C24	0.0287 (13)	0.0376 (13)	0.0441 (14)	−0.0059 (10)	0.0136 (10)	0.0052 (10)
C25	0.0309 (13)	0.0270 (11)	0.0373 (13)	−0.0047 (9)	0.0066 (10)	0.0020 (9)
C26	0.0261 (12)	0.0233 (10)	0.0252 (11)	0.0004 (8)	0.0037 (9)	0.0053 (8)
C27	0.0224 (11)	0.0267 (11)	0.0243 (11)	−0.0008 (8)	0.0039 (8)	0.0100 (8)
C28	0.0210 (12)	0.0428 (13)	0.0301 (12)	0.0020 (9)	0.0069 (9)	−0.0027 (9)
C29	0.0237 (13)	0.0299 (11)	0.0317 (12)	0.0022 (9)	0.0070 (9)	−0.0002 (9)
C30	0.0363 (15)	0.0685 (18)	0.0392 (14)	0.0008 (12)	−0.0072 (11)	0.0119 (12)
C31	0.0286 (12)	0.0246 (11)	0.0310 (12)	−0.0023 (9)	0.0040 (9)	−0.0010 (8)
C32	0.0277 (12)	0.0259 (11)	0.0230 (11)	0.0001 (9)	0.0028 (9)	−0.0053 (8)
C33	0.0245 (13)	0.0423 (13)	0.0337 (12)	−0.0021 (10)	0.0043 (9)	−0.0015 (10)
C34	0.0251 (13)	0.0438 (13)	0.0342 (13)	0.0066 (10)	−0.0006 (10)	0.0026 (10)
C35	0.0335 (13)	0.0331 (12)	0.0223 (11)	0.0020 (9)	−0.0010 (9)	0.0014 (9)
C36	0.0279 (12)	0.0281 (11)	0.0187 (10)	−0.0012 (9)	0.0038 (8)	−0.0056 (8)
C37	0.0235 (12)	0.0270 (11)	0.0222 (11)	0.0022 (8)	0.0009 (8)	−0.0058 (8)
C38	0.0336 (13)	0.0315 (12)	0.0300 (12)	0.0061 (9)	0.0099 (10)	−0.0004 (9)
C39	0.0349 (14)	0.0402 (13)	0.0462 (14)	0.0098 (10)	0.0058 (11)	0.0035 (11)
C40	0.0541 (19)	0.0661 (19)	0.0696 (19)	0.0306 (14)	0.0189 (15)	0.0068 (14)
C19'	0.027 (2)	0.040 (2)	0.015 (2)	0.0010 (16)	0.0051 (16)	0.0017 (16)
C20'	0.026 (2)	0.058 (2)	0.031 (2)	0.0047 (18)	0.0038 (16)	0.0048 (16)
O1	0.0213 (8)	0.0311 (8)	0.0285 (8)	−0.0024 (6)	0.0064 (6)	0.0033 (6)
O2	0.0285 (10)	0.1005 (14)	0.0341 (9)	0.0036 (9)	0.0076 (8)	0.0224 (9)
O3	0.0220 (9)	0.0618 (11)	0.0336 (9)	0.0012 (7)	0.0013 (7)	0.0087 (7)
O4	0.0218 (8)	0.0291 (8)	0.0294 (8)	0.0035 (6)	−0.0001 (6)	−0.0004 (6)
O5	0.0245 (8)	0.0312 (8)	0.0272 (8)	0.0039 (6)	0.0020 (6)	−0.0022 (6)
O7	0.0266 (9)	0.0423 (9)	0.0380 (9)	−0.0003 (7)	−0.0005 (7)	0.0187 (7)
O8	0.0233 (9)	0.0494 (10)	0.0688 (12)	−0.0035 (8)	0.0001 (8)	0.0252 (8)
O9	0.0202 (8)	0.0296 (7)	0.0251 (7)	0.0006 (6)	0.0031 (6)	0.0065 (6)

### Geometric parameters (Å, °)

**Table:**

C1—C36	1.514 (3)	C21—H21A	0.9700
C1—C2	1.516 (3)	C21—H21B	0.9700
C1—H1A	0.9700	C22—C23	1.386 (3)
C1—H1B	0.9700	C22—C27	1.398 (3)
C2—C3	1.388 (3)	C23—C24	1.384 (3)
C2—C7	1.392 (3)	C23—H23	0.9300
C3—C4	1.380 (3)	C24—C25	1.372 (3)
C3—H3	0.9300	C24—H24	0.9300
C4—C5	1.380 (3)	C25—C26	1.388 (3)
C4—H4	0.9300	C25—H25	0.9300
C5—C6	1.394 (3)	C26—C27	1.396 (3)
C5—H5	0.9300	C26—C31	1.515 (3)
C6—C7	1.392 (3)	C27—O1	1.388 (2)
C6—C11	1.514 (3)	C28—O1	1.417 (2)
C7—O9	1.395 (2)	C28—C29	1.512 (3)
C8—O9	1.419 (2)	C28—H28A	0.9700
C8—C9	1.498 (3)	C28—H28B	0.9700
C8—H8A	0.9700	C29—O2	1.190 (2)
C8—H8B	0.9700	C29—O3	1.328 (2)
C9—O8	1.194 (2)	C30—O3	1.443 (2)
C9—O7	1.329 (2)	C30—H30A	0.9600
C10—O7	1.443 (2)	C30—H30B	0.9600
C10—H10A	0.9600	C30—H30C	0.9600
C10—H10B	0.9600	C31—C32	1.516 (3)
C10—H10C	0.9600	C31—H31A	0.9700
C11—C12	1.512 (3)	C31—H31B	0.9700
C11—H11A	0.9700	C32—C33	1.387 (3)
C11—H11B	0.9700	C32—C37	1.393 (3)
C12—C13	1.392 (3)	C33—C34	1.378 (3)
C12—C17	1.394 (3)	C33—H33	0.9300
C13—C14	1.377 (3)	C34—C35	1.378 (3)
C13—H13	0.9300	C34—H34	0.9300
C14—C15	1.377 (3)	C35—C36	1.390 (3)
C14—H14	0.9300	C35—H35	0.9300
C15—C16	1.389 (3)	C36—C37	1.398 (3)
C15—H15	0.9300	C37—O5	1.392 (2)
C16—C17	1.393 (3)	C38—O5	1.427 (2)
C16—C21	1.514 (3)	C38—C39	1.504 (3)
C17—O4	1.399 (2)	C38—H38A	0.9700
C18—O4	1.432 (2)	C38—H38B	0.9700
C18—C19'	1.501 (4)	C39—C40	1.514 (3)
C18—C19	1.567 (7)	C39—H39A	0.9700
C18—H18A	0.9700	C39—H39B	0.9700
C18—H18B	0.9700	C40—H40A	0.9600
C18—H18C	0.9694	C40—H40B	0.9600
C18—H18D	0.9691	C40—H40C	0.9600
C19—C20	1.531 (10)	C19'—C20'	1.527 (5)
C19—H19A	0.9700	C19'—H19C	0.9700
C19—H19B	0.9700	C19'—H19D	0.9700
C20—H20A	0.9600	C20'—H20D	0.9600
C20—H20B	0.9600	C20'—H20E	0.9600
C20—H20C	0.9600	C20'—H20F	0.9600
C21—C22	1.514 (3)		
			
C36—C1—C2	111.84 (15)	H21A—C21—H21B	107.9
C36—C1—H1A	109.2	C23—C22—C27	118.06 (18)
C2—C1—H1A	109.2	C23—C22—C21	121.86 (18)
C36—C1—H1B	109.2	C27—C22—C21	120.04 (18)
C2—C1—H1B	109.2	C24—C23—C22	121.24 (19)
H1A—C1—H1B	107.9	C24—C23—H23	119.4
C3—C2—C7	118.10 (18)	C22—C23—H23	119.4
C3—C2—C1	121.28 (18)	C25—C24—C23	119.5 (2)
C7—C2—C1	120.51 (18)	C25—C24—H24	120.3
C4—C3—C2	121.13 (19)	C23—C24—H24	120.3
C4—C3—H3	119.4	C24—C25—C26	121.7 (2)
C2—C3—H3	119.4	C24—C25—H25	119.2
C5—C4—C3	119.31 (19)	C26—C25—H25	119.2
C5—C4—H4	120.3	C25—C26—C27	117.82 (18)
C3—C4—H4	120.3	C25—C26—C31	120.29 (18)
C4—C5—C6	121.87 (19)	C27—C26—C31	121.50 (18)
C4—C5—H5	119.1	O1—C27—C26	121.21 (17)
C6—C5—H5	119.1	O1—C27—C22	116.65 (17)
C7—C6—C5	117.16 (18)	C26—C27—C22	121.58 (18)
C7—C6—C11	121.91 (18)	O1—C28—C29	114.66 (16)
C5—C6—C11	120.93 (17)	O1—C28—H28A	108.6
C6—C7—C2	122.27 (18)	C29—C28—H28A	108.6
C6—C7—O9	119.21 (17)	O1—C28—H28B	108.6
C2—C7—O9	118.48 (17)	C29—C28—H28B	108.6
O9—C8—C9	109.68 (16)	H28A—C28—H28B	107.6
O9—C8—H8A	109.7	O2—C29—O3	123.88 (19)
C9—C8—H8A	109.7	O2—C29—C28	126.29 (19)
O9—C8—H8B	109.7	O3—C29—C28	109.79 (17)
C9—C8—H8B	109.7	O3—C30—H30A	109.5
H8A—C8—H8B	108.2	O3—C30—H30B	109.5
O8—C9—O7	124.08 (19)	H30A—C30—H30B	109.5
O8—C9—C8	127.12 (18)	O3—C30—H30C	109.5
O7—C9—C8	108.80 (17)	H30A—C30—H30C	109.5
O7—C10—H10A	109.5	H30B—C30—H30C	109.5
O7—C10—H10B	109.5	C26—C31—C32	110.78 (16)
H10A—C10—H10B	109.5	C26—C31—H31A	109.5
O7—C10—H10C	109.5	C32—C31—H31A	109.5
H10A—C10—H10C	109.5	C26—C31—H31B	109.5
H10B—C10—H10C	109.5	C32—C31—H31B	109.5
C12—C11—C6	113.08 (15)	H31A—C31—H31B	108.1
C12—C11—H11A	109.0	C33—C32—C37	118.33 (18)
C6—C11—H11A	109.0	C33—C32—C31	119.97 (18)
C12—C11—H11B	109.0	C37—C32—C31	121.56 (18)
C6—C11—H11B	109.0	C34—C33—C32	121.0 (2)
H11A—C11—H11B	107.8	C34—C33—H33	119.5
C13—C12—C17	117.29 (18)	C32—C33—H33	119.5
C13—C12—C11	121.17 (18)	C35—C34—C33	119.8 (2)
C17—C12—C11	121.49 (17)	C35—C34—H34	120.1
C14—C13—C12	121.53 (19)	C33—C34—H34	120.1
C14—C13—H13	119.2	C34—C35—C36	121.37 (19)
C12—C13—H13	119.2	C34—C35—H35	119.3
C13—C14—C15	119.73 (19)	C36—C35—H35	119.3
C13—C14—H14	120.1	C35—C36—C37	117.75 (18)
C15—C14—H14	120.1	C35—C36—C1	120.98 (18)
C14—C15—C16	121.12 (19)	C37—C36—C1	121.01 (17)
C14—C15—H15	119.4	O5—C37—C32	118.83 (17)
C16—C15—H15	119.4	O5—C37—C36	119.34 (17)
C15—C16—C17	117.85 (18)	C32—C37—C36	121.67 (18)
C15—C16—C21	119.51 (18)	O5—C38—C39	108.81 (17)
C17—C16—C21	122.59 (17)	O5—C38—H38A	109.9
C16—C17—C12	122.25 (17)	C39—C38—H38A	109.9
C16—C17—O4	119.35 (17)	O5—C38—H38B	109.9
C12—C17—O4	118.39 (17)	C39—C38—H38B	109.9
O4—C18—C19'	113.0 (2)	H38A—C38—H38B	108.3
O4—C18—C19	101.7 (3)	C38—C39—C40	112.44 (19)
C19'—C18—C19	28.5 (3)	C38—C39—H39A	109.1
O4—C18—H18A	111.4	C40—C39—H39A	109.1
C19'—C18—H18A	83.0	C38—C39—H39B	109.1
C19—C18—H18A	111.5	C40—C39—H39B	109.1
O4—C18—H18B	111.4	H39A—C39—H39B	107.8
C19'—C18—H18B	124.8	C39—C40—H40A	109.5
C19—C18—H18B	111.3	C39—C40—H40B	109.5
H18A—C18—H18B	109.3	H40A—C40—H40B	109.5
O4—C18—H18C	109.0	C39—C40—H40C	109.5
C19'—C18—H18C	109.1	H40A—C40—H40C	109.5
C19—C18—H18C	136.7	H40B—C40—H40C	109.5
H18A—C18—H18C	28.2	C18—C19'—C20'	110.2 (3)
H18B—C18—H18C	85.2	C18—C19'—H19C	109.6
O4—C18—H18D	108.9	C20'—C19'—H19C	109.6
C19'—C18—H18D	108.9	C18—C19'—H19D	109.6
C19—C18—H18D	89.5	C20'—C19'—H19D	109.6
H18A—C18—H18D	128.6	H19C—C19'—H19D	108.1
H18B—C18—H18D	23.7	C19'—C20'—H20D	109.5
H18C—C18—H18D	107.9	C19'—C20'—H20E	109.5
C20—C19—C18	106.3 (5)	H20D—C20'—H20E	109.5
C20—C19—H19A	110.5	C19'—C20'—H20F	109.5
C18—C19—H19A	110.5	H20D—C20'—H20F	109.5
C20—C19—H19B	110.5	H20E—C20'—H20F	109.5
C18—C19—H19B	110.5	C27—O1—C28	120.32 (15)
H19A—C19—H19B	108.7	C29—O3—C30	115.85 (17)
C22—C21—C16	111.84 (15)	C17—O4—C18	110.87 (14)
C22—C21—H21A	109.2	C37—O5—C38	114.62 (14)
C16—C21—H21A	109.2	C9—O7—C10	116.94 (16)
C22—C21—H21B	109.2	C7—O9—C8	112.20 (14)
C16—C21—H21B	109.2		
			
C36—C1—C2—C3	113.1 (2)	C31—C26—C27—O1	−3.2 (3)
C36—C1—C2—C7	−63.0 (2)	C25—C26—C27—C22	−4.8 (3)
C7—C2—C3—C4	1.9 (3)	C31—C26—C27—C22	167.99 (17)
C1—C2—C3—C4	−174.32 (18)	C23—C22—C27—O1	175.26 (17)
C2—C3—C4—C5	1.4 (3)	C21—C22—C27—O1	−2.5 (3)
C3—C4—C5—C6	−2.1 (3)	C23—C22—C27—C26	3.7 (3)
C4—C5—C6—C7	−0.5 (3)	C21—C22—C27—C26	−174.02 (17)
C4—C5—C6—C11	178.82 (18)	O1—C28—C29—O2	35.9 (3)
C5—C6—C7—C2	3.9 (3)	O1—C28—C29—O3	−146.37 (17)
C11—C6—C7—C2	−175.35 (17)	C25—C26—C31—C32	105.4 (2)
C5—C6—C7—O9	−178.60 (16)	C27—C26—C31—C32	−67.2 (2)
C11—C6—C7—O9	2.1 (3)	C26—C31—C32—C33	−54.8 (2)
C3—C2—C7—C6	−4.6 (3)	C26—C31—C32—C37	120.8 (2)
C1—C2—C7—C6	171.58 (17)	C37—C32—C33—C34	−0.7 (3)
C3—C2—C7—O9	177.88 (16)	C31—C32—C33—C34	175.00 (18)
C1—C2—C7—O9	−5.9 (3)	C32—C33—C34—C35	−1.2 (3)
O9—C8—C9—O8	−6.0 (3)	C33—C34—C35—C36	1.2 (3)
O9—C8—C9—O7	173.24 (15)	C34—C35—C36—C37	0.8 (3)
C7—C6—C11—C12	61.6 (2)	C34—C35—C36—C1	−173.37 (18)
C5—C6—C11—C12	−117.6 (2)	C2—C1—C36—C35	111.4 (2)
C6—C11—C12—C13	−109.1 (2)	C2—C1—C36—C37	−62.6 (2)
C6—C11—C12—C17	68.2 (2)	C33—C32—C37—O5	178.23 (16)
C17—C12—C13—C14	−1.4 (3)	C31—C32—C37—O5	2.6 (3)
C11—C12—C13—C14	175.98 (18)	C33—C32—C37—C36	2.8 (3)
C12—C13—C14—C15	−2.3 (3)	C31—C32—C37—C36	−172.84 (17)
C13—C14—C15—C16	2.6 (3)	C35—C36—C37—O5	−178.24 (16)
C14—C15—C16—C17	0.9 (3)	C1—C36—C37—O5	−4.1 (3)
C14—C15—C16—C21	−176.59 (18)	C35—C36—C37—C32	−2.9 (3)
C15—C16—C17—C12	−4.9 (3)	C1—C36—C37—C32	171.33 (17)
C21—C16—C17—C12	172.51 (17)	O5—C38—C39—C40	−172.66 (19)
C15—C16—C17—O4	176.45 (16)	O4—C18—C19'—C20'	61.2 (3)
C21—C16—C17—O4	−6.2 (3)	C19—C18—C19'—C20'	−10.4 (6)
C13—C12—C17—C16	5.1 (3)	C26—C27—O1—C28	−75.8 (2)
C11—C12—C17—C16	−172.29 (17)	C22—C27—O1—C28	112.63 (19)
C13—C12—C17—O4	−176.18 (16)	C29—C28—O1—C27	−61.1 (2)
C11—C12—C17—O4	6.4 (3)	O2—C29—O3—C30	4.0 (3)
O4—C18—C19—C20	177.9 (5)	C28—C29—O3—C30	−173.75 (18)
C19'—C18—C19—C20	−65.2 (7)	C16—C17—O4—C18	−85.1 (2)
C15—C16—C21—C22	65.0 (2)	C12—C17—O4—C18	96.18 (19)
C17—C16—C21—C22	−112.3 (2)	C19'—C18—O4—C17	172.2 (2)
C16—C21—C22—C23	−110.7 (2)	C19—C18—O4—C17	−160.3 (4)
C16—C21—C22—C27	67.0 (2)	C32—C37—O5—C38	96.2 (2)
C27—C22—C23—C24	−0.5 (3)	C36—C37—O5—C38	−88.3 (2)
C21—C22—C23—C24	177.19 (19)	C39—C38—O5—C37	−173.18 (17)
C22—C23—C24—C25	−1.5 (3)	O8—C9—O7—C10	−2.8 (3)
C23—C24—C25—C26	0.4 (3)	C8—C9—O7—C10	177.92 (17)
C24—C25—C26—C27	2.7 (3)	C6—C7—O9—C8	92.3 (2)
C24—C25—C26—C31	−170.17 (19)	C2—C7—O9—C8	−90.1 (2)
C25—C26—C27—O1	−175.94 (16)	C9—C8—O9—C7	171.14 (16)

### Hydrogen-bond geometry (Å, °)

**Table:**

*D*—H···*A*	*D*—H	H···*A*	*D*···*A*	*D*—H···*A*
C30—H30B···O8^i^	0.96	2.58	3.406 (3)	144
C31—H31B···O5	0.97	2.40	2.859 (2)	109
C24—H24···O3^ii^	0.93	2.58	3.309 (3)	135
C21—H21A···O4	0.97	2.45	2.899 (2)	108
C21—H21A···O2	0.97	2.56	3.255 (3)	129
C10—H10C···O8^iii^	0.96	2.59	3.510 (3)	161

Symmetry codes: (i) *x*+1, −*y*+3/2, *z*+1/2; (ii) *x*−1, *y*, *z*; (iii) −*x*, −*y*+2, −*z*.
